# HIF-3α beyond the dominant-negative paradigm: Isoform diversity, species divergence, and redox-driven diseases

**DOI:** 10.1016/j.redox.2026.104464

**Published:** 2026-10-31

**Authors:** Zahra Bint e Sohail, Thomas Kietzmann, Daniela Mennerich

**Affiliations:** Faculty of Biochemistry and Molecular Biology, Biocenter Oulu, University of Oulu, 90220, Oulu, Finland

**Keywords:** Hypoxia, Redox regulation, Reactive oxygen species, HIF-3, Cancer, Diabetes, Cardiac diseases

## Abstract

Hypoxia-inducible factor 3 alpha (HIF-3α) has long been considered a minor, inhibitory branch of the HIF pathway. However, accumulating evidence suggests that HIF-3α is a context-dependent regulator with transcriptional activator and dominant-negative functions. The human *HIF3A* locus undergoes extensive alternative splicing, yielding at least eight to ten protein isoforms. In contrast, the mouse *Hif3a* gene produces only three major variants. This creates substantial species divergence in isoform architecture and expression. Full-length human isoforms, such as HIF-3α1, HIF-3α2, and HIF-3α9, are bona fide transcription factors that regulate both canonical and non-canonical hypoxic targets, including EPO, LC3C, REDD1, SQRDL, and IGFBP-1. In contrast, truncated variants, such as HIF-3α4, antagonize HIF-1/2-dependent angiogenic and metabolic programs in a tissue-specific manner. Mouse models demonstrate that canonical HIF-3α integrates developmental and redox cues in the lungs, heart, and metabolism. Furthermore, human *HIF3A* methylation and isoform-specific expression have been linked to obesity, liver dysfunction, diabetes, chronic lung disease, neurodegeneration, and cancer. Full-length isoforms often promote tumor growth and metastasis (e.g., in colorectal and pancreatic cancers), whereas truncated isoforms constrain angiogenesis or stabilize oncogenic HIF-2α in certain contexts. In this review, we integrate these findings into an isoform-resolved, cross-species framework that distinguishes experimentally validated HIF-3α functions from indirect associations and proposed redox mechanisms. We propose that HIF-3α acts less as a single hypoxia transcription factor and more as an isoform-specific rheostat that shapes acute, chronic, and redox-stressed states. Finally, we outline the need for long-read transcriptomics, isoform-specific assays, evidence-stratified disease models, and targeted modulation of individual HIF-3α variants to translate this complexity into therapeutic strategies for redox-driven human diseases.

## 1 Introduction

Oxygen (O_2_) is a diradical molecule with two unpaired electrons in its ground state. The complete four-electron reduction of O_2_ to water (H_2_O) in the mitochondrial electron transport chain (ETC) enables efficient ATP production through oxidative phosphorylation. However, during the electron transfer process, incomplete reduction at complexes I and III, where single-electron transfers occur, can lead to the generation of reactive oxygen species (ROS) including superoxide (O_2_•^-^), hydrogen peroxide (H_2_O_2_), and hydroxyl radicals (•OH), creating a fundamental redox challenge for aerobic organisms [1], [2]. This dual nature of oxygen-which is essential for energy metabolism yet capable of generating potentially damaging ROS, has driven the evolution of sophisticated oxygen-sensing mechanisms, such as the hypoxia-inducible factor (HIF) pathway, alongside dedicated ROS scavenging systems, primarily regulated by the NRF2 pathway[3].

The HIF pathway represents a primary intracellular transcriptional oxygen-sensing mechanism in metazoans. While other oxygen-sensing mechanisms exist (such as the carotid body for systemic oxygen sensing), the HIF pathway is the dominant transcriptional regulatory system for cellular adaptation to hypoxia across most metazoan tissues. The HIF family comprises of three rather labile HIFα paralogs known as HIF-1α, HIF-2α, and HIF-3α that dimerize with the constitutively expressed HIF-1β subunit [4], [5].

While HIF-1α and HIF-2α function primarily as transcriptional activators of hypoxic adaptation genes, HIF-3α represents a distinct regulatory branch with unique properties that extend beyond classical oxygen sensing [6], [7]. Unlike its paralogs, HIF-3α functions primarily as a modulator of the hypoxic response, with its activity shaped by extensive alternative splicing generating numerous isoforms with divergent functions.

Emerging evidence suggests that HIF-3α plays a significant role in redox signaling pathways, though the molecular mechanisms underlying this relationship remain less characterized than those of HIF-1α [8], [9]. While HIF-1α′s redox connections-including ROS-mediated stabilization and regulation of select antioxidant responses are well-established [10], [11], [12], [13], the specific redox functions of HIF-3α represent an active area of investigation with important implications for understanding its tissue-specific roles in human disease.

A critical but often overlooked aspect of HIF-3α biology is the substantial evolutionary divergence between human and mouse isoform complexity [14]. While the mouse *Hif3a* gene generates three major isoforms (canonical HIF-3α, NEPAS, and IPAS), the human *HIF3A* locus produces at least 19 splice variants, with 10 experimentally confirmed protein isoforms exhibiting tissue-specific expression patterns [15]. This species-specific complexity has profound implications for translating findings from murine models to human pathophysiology.

Currently, two limitations exist in the field. First, many studies measure total HIF3A mRNA or total HIF-3α protein without resolving the isoform that is functionally active, and immunohistochemical data may not distinguish HIF-3α1, HIF-3α2, HIF-3α4, or HIF-3α9. Second, the redox dimension of HIF-3α biology is often inferred from the well-characterized redox regulation of HIF-1α and HIF-2α, whereas direct redox modification of HIF-3α remains limited. To address these gaps, this review separates experimentally validated mechanisms from correlative observations and from models that require further investigations. It also adds a post-transcriptional layer, including microRNA and lncRNA control of HIF3A isoforms, and uses an evidence hierarchy to compare physiological and pathological findings.

This review provides the first comprehensive analysis of HIF-3α through the dual lenses of redox biology and cross-species comparison. We systematically examine how HIF-3α′s unique structural features, particularly its leucine zipper domain and absence of a C-terminal transactivation domain, enable its specialized functions. By contrasting human and mouse isoform expression, regulation, and function, we highlight both conserved mechanisms and species-specific adaptations that inform our understanding of HIF-3α in human disease.

## 2 HIF-1α and HIF-2α isoforms, domain organization and canonical versus non-canonical regulation

### 2.1 HIF-1α

In humans, the *HIF1A* gene is localized on chromosome 14q23.2 (GRCh38 assembly). It spans about 53 kB and includes 15 exons [16]. Four protein coding isoforms of HIF-1α have been described so far. Isoform HIF1A-202 (826 residues) is the canonical and most abundant, encoding a basic helix-loop-helix (bHLH)-PER-ARNT-SIM (PAS) domain-containing protein. HIF1A-201 encodes a variant with 735 amino acids due to skipping of exon 14 which causes a frameshift and premature stop codon, producing a variant that lacks the C-terminal transactivation and inhibitory domains. HIF1A-203 encodes a variant with 827 amino acids that arises from use of an alternative 3′ splice acceptor site within intron 1 (exon 1-exon 2 junction), producing a 3-nucleotide insertion and two amino-acid substitutions N-terminal of the bHLH domain. HIF1A-204 encodes a protein with 850 amino acids. The latter isoform has weaker transcriptional activity compared to the canonical HIF-1α isoform but appears to be an activation-inducible mRNA isoform analogous to that expressed from the mouse I.1 mRNA since it is upregulated in activated peripheral T lymphocytes [17].

The mouse *Hif1a* gene is located on chromosome 12 and contains 15 exons, or 16 when the two alternative first exons (I.1 and I.2) are counted separately, spanning about 46 kb (RefSeq GRCm39). Three cDNA sequences encoding proteins of 836, 822, and 810 amino acids resulting from alternative splicing have been reported [18], [19]. Additionally, splicing of an alternative upstream exon I.1 to exon 2 utilizes a methionine codon in exon 2 (rather than exon 1) for translation initiation. As a result, the I.1 mRNA encodes a protein that lacks 12 N-terminal amino acid residues [20], [21], [22]. Although no functional differences among these HIF-1α isoforms have been definitively established, the I.1/I.2 promoter-driven isoforms do differ functionally: T-cell receptor (TCR)-mediated signaling selectively induces the HIF-1α I.1 mRNA isoform, which follows a pattern of immediate early response genes. Genetic deletion of the TCR-inducible isoform I.1 of HIF-1α in mice results in significant enhancement of T-cell functions [23].

### 2.2 HIF-2α

The human *EPAS1* gene encoding HIF-2α is localized on chromosome 2p21 and the mouse *Epas1* gene is located on chromosome 17; both include 16 exons encoding for a canonical bHLH-PAS protein of 870 and 874 amino acids, respectively [24]. According to the ENSEMBL genome browser, the human *EPAS1* gene has ten splice variants, two protein-coding, and eight non-coding. The canonical protein-coding transcript EPAS1-201 encodes an 870 amino acid protein, while the shorter protein-coding transcript EPAS1-202 is derived from seven exons and encodes a protein with 259 amino acids. Non-coding transcripts are either processed transcripts lacking an open reading frame or transcripts with retained introns [25].

### 2.3 Protein domains and homology

The HIF-1α and HIF-2α paralogous exhibit 48% amino acid sequence identity overall, as determined by sequence alignment in humans and mice [26]. Domain analysis reveals 83-85% identity in the bHLH domain, facilitating binding to HRE sequences (5′-RCGTG-3′), and 70-74% in PAS-A/B/PAC domains, enabling ARNT dimerization but differing in coactivator affinity [26], [27]. This conservation supports shared hypoxic signaling while allowing specificity. HIF-1α activates glycolytic targets via p300/CBP, while HIF-2α engages ETS-1 for erythropoiesis genes like EPO and VEGFR2 (Flk-1) [26]. The ODDD contains prolines hydroxylated by PHDs (HIF-1α: P402/P564; HIF-2α: P405/P531), overlapping N-TAD, with ID separating from C-TAD asparagine (HIF-1α: N803; HIF-2α: N847) targeted by FIH [27]. HIF-2α′s G537 insertion has been shown to reduce PHD2 binding, thereby enhancing stability [27], (Fig. 1).

**Fig. 1:**
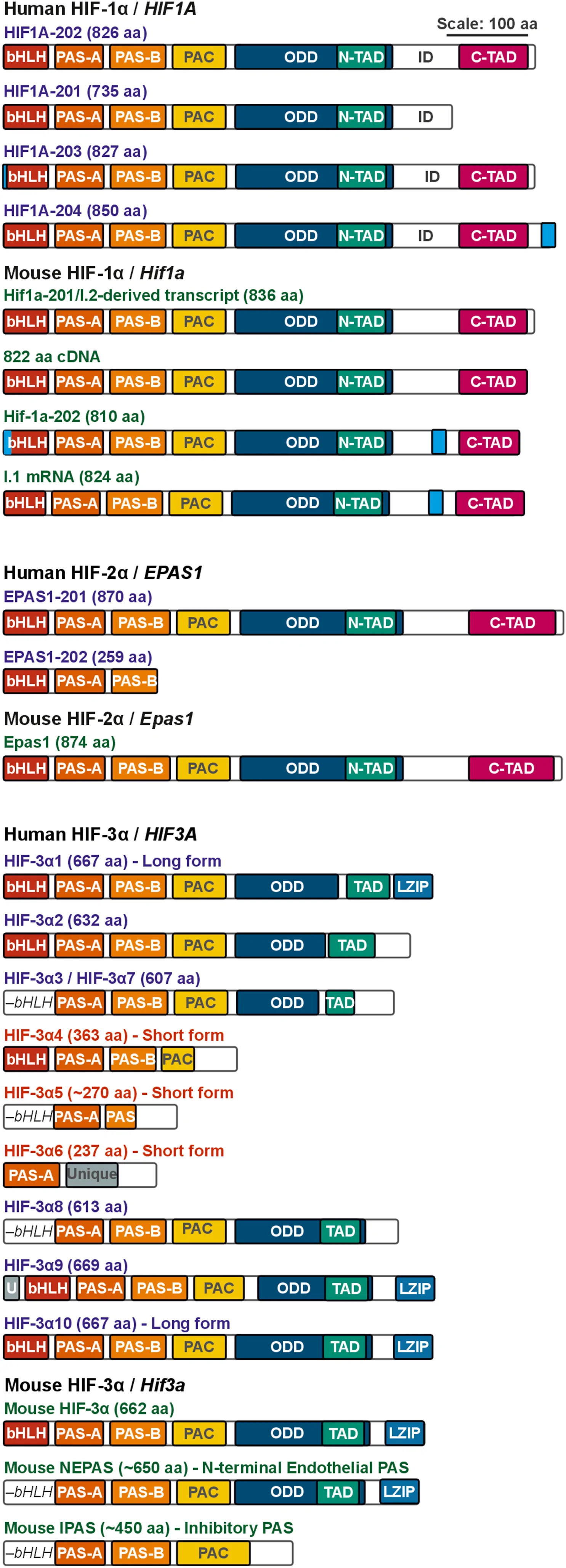
**Domain organization of mammalian HIF-α proteins, with emphasis on HIF-3α splice variants.** Protein products are drawn to a common amino-acid scale. The key conserved domains correspond to basic helix-loop-helix (bHLH), Per-ARNT-Sim (PAS A/B), the PAS-associated C-terminal motif (PAC), the oxygen dependent degradation domain (ODD), N-terminal (N-TAD) and C-terminal (C-TAD) exhibited by HIF-1α and HIF-2α. HIF-3α differs markedly, lacking a C-terminal (C-TAD) and instead possessing a unique leucine zipper (LZIP) domain in some variants.

### 2.4 Canonical regulation

The canonical, oxygen-dependent regulation of HIF-1α and HIF-2α is primarily realized through protein stability control rather than mRNA regulation. Under normoxic conditions, HIFα subunits are hydroxylated by prolyl hydroxylase domain enzymes (PHDs), most prominently PHD2 (also known as *EGLN1*), on conserved proline residues within the ODDD [28]. This hydroxylation enables recognition by the von Hippel-Lindau protein (pVHL), leading to polyubiquitination and proteasomal degradation of HIF-1α and HIF-2α. Additionally, the asparaginyl hydroxylase factor-inhibiting HIF (FIH) modifies HIF-1α and HIF-2α on their C-TAD, inhibiting interaction with coactivators like CBP/p300 and reducing transactivation [29], [30]. Although the catalytic activity of both PHDs and FIH is oxygen-dependent, FIH, due to its higher Km for oxygen (approximately 100 μM), is less sensitive to oxygen availability than PHDs [28], (Fig. 2).

**Fig. 2:**
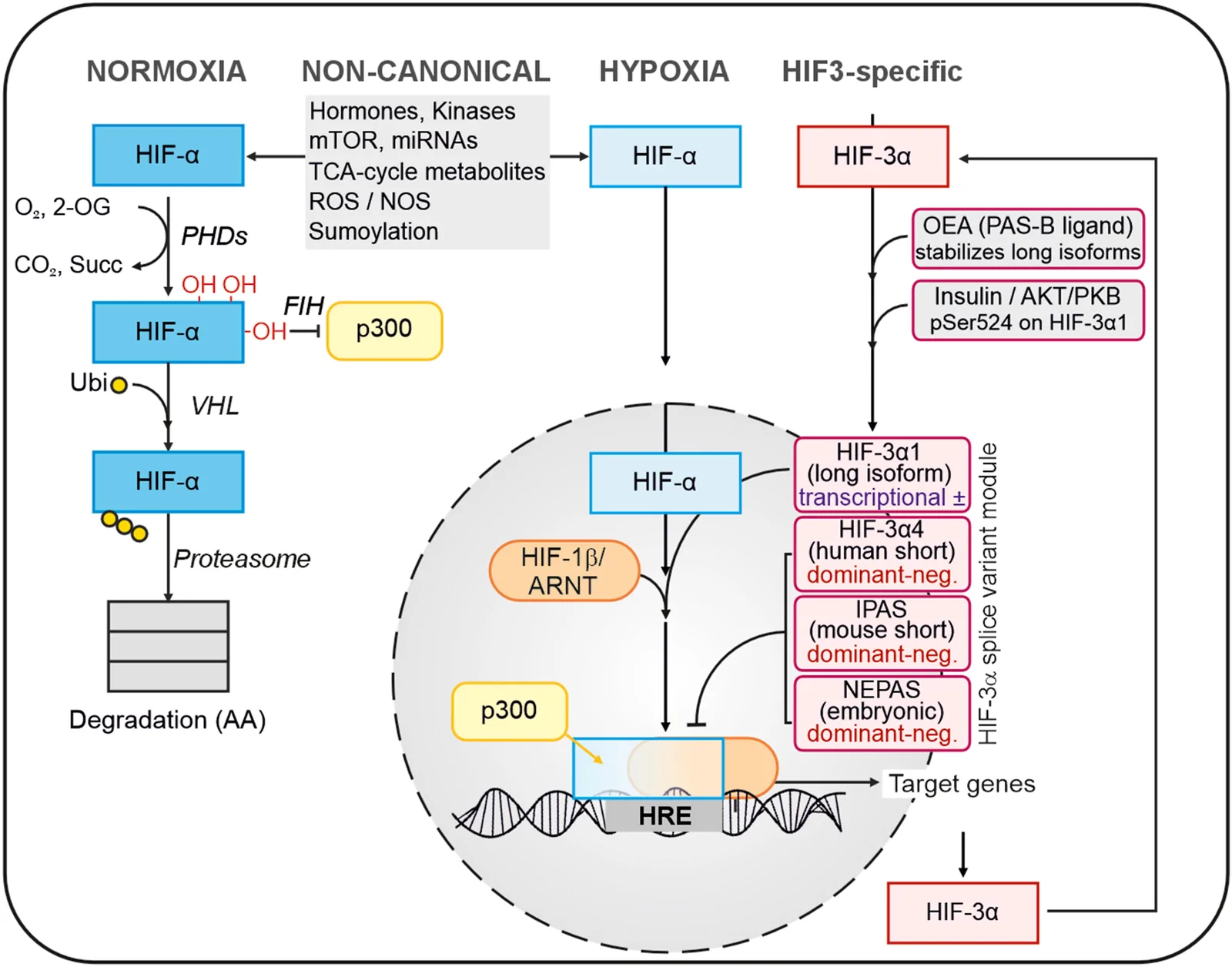
**Oxygen-dependent regulation of HIFs and HIF-3α-specific regulatory mechanisms.** The scheme summarizes canonical normoxic HIF-α subunit degradation, its modulation by non-hypoxic inputs, including hormones, kinases, ROS/NOS, miRNAs, metabolites, mTOR, and post-translational modifications, e.g., sumoylation. Hypoxia stabilizes HIFα, promotes nuclear translocation and HIFα -ARNT dimerization. Alternatively spliced HIF-3α variants can undergo-specific regulation, e.g., via OEA binding to PAS-B or AKT/PKB-mediated phosphorylation of HIF-3α1 at Ser524. Transcriptionally competent HIF-3α variants can associate with ARNT/HIF-1β, bind HREs and regulate HIF-3α-responsive genes. Shorter splice products of the *HIF3A* locus, such as HIF-3α4 appear to interact with HIF-1α to form non-productive complexes that prevent productive HIF-1α–ARNT association and HRE-dependent transcription, thereby acting as a dominant-negative regulators. The scheme provides a simplified overview; the cellular consequences of HIF-3α regulation depend on the expressed HIF-3α isoform, cellular context, and environmental conditions. **Abbreviations:** 2-OG, 2-oxoglutarate; AKT/PKB, protein kinase B; ARNT, aryl hydrocarbon receptor nuclear translocator; FIH, factor inhibiting HIF; HIF, hypoxia-inducible factor; HRE, hypoxia-response element; IPAS, inhibitory PAS-domain protein; OEA, oleoylethanolamide; PAS-B, Per–ARNT–Sim B domain; PHD, prolyl hydroxylase domain protein; pVHL, von Hippel-Lindau protein; ROS/NOS, reactive oxygen and nitrogen species; Ubi, ubiquitin.

During hypoxia, the activity of PHDs and FIH is suppressed, preventing HIFα hydroxylation. This allows HIFα subunits to accumulate, translocate to the nucleus, form heterodimers with HIF-1β, and bind to hypoxia response elements (HREs) in target genes [31]. The HIF-1β/HIF-α heterodimer recruits coactivators such as p300 and SRC-1, enhancing transcriptional activity by facilitating chromatin remodeling and recruitment of RNA polymerase II to HIF target gene promoters [32].

### 2.5 Non-canonical regulation

Non-canonical regulation of HIF-α comprises oxygen-independent inputs that modulate HIF abundance or transactivation outside the PHD-pVHL-proteasome axis. Mitochondrial/NADPH-oxidase-derived ROS can stabilize HIF-1α by impairing PHD catalysis via redox modification of PHD2 [33] and by localized ER-proximal Fenton chemistry that influences hydroxylase activity and co-activator recruitment [34]. Direct redox modifications of HIF-1α itself further contribute: S-nitrosylation at Cys 533 (mouse; equivalent to human Cys520) in the ODD prevents pVHL binding and stabilizes the protein in response to nitric oxide [35]. Similarly, S-glutathionylation at the same Cys520 residue under oxidative/ischemic stress blocks VHL binding [36]. Redox regulation of Cys 800 in the C-TAD controls p300/CBP recruitment [37]. In addition, ROS also drive NF-κB/ERK and PI3K signaling to increase *HIF1A* transcription and support translation under normoxia [12], [38]. Similar, though less extensively characterized, redox-sensitive mechanisms operate in HIF-2α.

Metabolic rewiring provides further oxygen independent control. Succinate accumulation in SDH deficiency competitively inhibits 2 oxoglutarate–dependent PHDs, stabilizing HIFα in normoxia [39]. In FH deficiency, fumarate promotes a pseudohypoxic program and also drives HIF independent signaling (e.g., KEAP1 succination/Nrf2 activation) [40]. Further oncometabolites such as hydroxyglutarate can differentially tune the pathway, with L2-hydroxyglutarate promoting and D2-hydroxyglutarate suppressing HIF-1α activity in a context and enantiomer specific manner [40], [41].

Nitric oxide (NO) exerts concentration- and context -dependent effects on HIF: acutely, NO can stabilize HIF-1α by inhibiting PHDs, whereas sustained NO induces PHD2 and accelerates HIF-1α degradation-a biphasic behavior mechanistically traced to HIF-1-dependent PHD2 upregulation [42].

Growth-factor and nutrient signaling fine-tune HIF at multiple levels. PI3K-AKT-mTOR signaling augments HIF-1 activity and synthesis through direct coupling of RAPTOR to an HIF-1α TOS motif and rapamycin-sensitive control [43], consistent with earlier evidence that mTOR promotes HIF-1α de novo synthesis through 4*E*-BP1/eIF4E-dependent translation [44]. Further, MAPK/ERK signaling supports nuclear transactivation, whereas GSK3β-dependent phosphorylation creates an FBXW7-recognized phosphodegron that promotes VHL-independent HIF-1α degradation, antagonized by USP28 [45], [46].

Co-activator control further modulates HIFs. The HIF-1α C-terminal activation domain engages p300/CBP for transactivation, and p300-dependent acetylation can increase HIF-1α stability and output [47], [48]. Post-translational methylation adds another oxygen-independent layer: HIF-1α is methylated by SET7/9, promoting its degradation, whereas LSD1 removes this mark and stabilizes HIF-1α; LSD1 also stabilizes HIF-1α by demethylating RACK1 and suppressing oxygen-independent turnover [49], [50]. SUMOylation influences HIF signaling in a context-dependent manner, with recent data indicating predominantly indirect repression of HIF-1α stability and transactivation by global SUMOylation, although cell-type-specific studies have reported SUMO-associated increases in HIF-1α function [51], [52].

Inflammatory mediators interconnect with HIF at the transcriptional level: TNF-α/IL-1β–driven NF-κB binds a functional element in the *HIF1A* promoter and upregulates HIF-1α mRNA under normoxia and hypoxia, thereby coupling inflammatory signaling to hypoxia responses [38], [53].

Clock control intersects with hypoxia signaling with rhythmic oxygen levels resetting clocks via HIF-1α and interaction of clock proteins such as BMAL1/CLOCK and CRY1 coordinate hypoxic responses with metabolic rhythms [54], [55]. CRY1 directly binds HIF-1α and HIF-2α, reduces HIF-1α half-life, and limits DNA binding, thereby imposing circadian constraints on HIF activity [56].

Epigenetic remodeling adds further oxygen-independent complexity. Hypoxia suppresses TET dioxygenases and globally reduces 5-hydroxymethylcytosine, driving promoter hypermethylation independent of HIF-α stabilization [57].

Non-coding RNAs integrate hypoxic and inflammatory inputs with HIF control. The hypoxamir miR-210 is a direct HIF target that remodels mitochondrial metabolism by repressing ISCU, thereby reinforcing hypoxia signaling [58], [59]. MiR-155, induced by angiogenic and inflammatory stimuli, modulates HIF-1α expression and endothelial maturation in a context-dependent manner [60].

Collectively, these oxygen-independent inputs-redox and NO signaling, oncometabolites, kinase and co-activator control, methylation/SUMOylation, inflammatory and circadian cues, epigenetic remodeling, and microRNAs-explain robust HIF activity in normoxic yet stressed tissues and offer therapeutic leverage points beyond the PHD-pVHL axis (Fig. 2).

When HIF-3α is discussed in redox terms, this distinction is essential. The mechanisms above are experimentally established for HIF-1α and, to a lesser extent, HIF-2α. For HIF-3α, they should be treated as a comparative framework unless a HIF-3α-specific experiment is cited.

## 3 HIF-1α and HIF-2α: similar but distinct regulators of the hypoxia response paving the way for HIF-3α

Both HIF-1α and HIF-2α coordinate cellular adaptation to reduced oxygen within a shared framework involving heterodimerization with HIF-1β/ARNT, recruitment of CBP/p300, and binding of HREs, though they have non-redundant biology [61], [62], [63].

HIF-1α is ubiquitously expressed in all tissues, while HIF-2α expression is more limited to specific cell types (e.g., endothelium, type II pneumocytes, interstitial kidney cells), consistent with genetic and physiological studies [26], [62]. Pan-genomic ChIP-seq shows HIF-1α is promoter-proximal (∼40% sites within 2.5 kb of TSS), whereas HIF-2α preferentially occupies promoter-distal enhancer-like sites (∼20% near TSS). These isoform-specific distributions persist across cell types and degrees/durations of hypoxia, and analyses in HIF-1α/2α knockout models indicate limited compensation of one isoform for the other's binding [64]. This promoter-vs-enhancer bias has also been replicated in primary human macrophages and in earlier HIF cistrome maps [64]. Concordant with these binding preferences, HIF-1α sites are enriched in promoter-associated chromatin (e.g., H3K4me3/H3K9ac), whereas HIF-2α sites are enriched in enhancer-associated chromatin (e.g., H3K4me1/H3K27ac); HIF occupancy is favored at accessible DNase-hypersensitive regions, consistent with a dependency on pre-established chromatin accessibility [32], [64], [65], [66].

Transcriptional and genetic evidence supports a division of labor whereby HIF-1α dominates acute hypoxic adaptation, prominently inducing glycolysis (e.g., PGK1, LDHA) and cell-death programs such as BNIP3, while HIF-2α contributes more to prolonged/chronic hypoxia, including erythropoiesis (EPO), vascular remodeling/angiogenesis, and metabolic rewiring [63], [67], [68] These binding distinctions, coupled with differences in their N-terminal transactivation domains (N-TADs), contribute to their differential regulation under acute and chronic hypoxia, shaping distinct but partially overlapping transcriptional programs. HIF-1α orchestrates the acute hypoxic response by promoting glycolysis through genes like PGK1 and LDHA and triggering apoptotic pathways via BNIP3. In contrast, HIF-2α drives chronic hypoxia adaptation by regulating erythropoiesis (EPO), angiogenesis (VEGF), lipid metabolism (ADRP), and immune evasion, the latter achieved through suppression of macrophage phagocytosis via MARCO downregulation and enhancement of stemness by upregulating OCT-4 [64], [65].

## 4 HIF3A isoform architecture in mouse and human

Mouse *Hif3a* and human *HIF3A* loci encode related but non-identical isoform repertoires. The mouse locus produces canonical HIF-3α, NPAS, and IPAS, whereas the human locus produces multiple long, short, and tissue-restricted variants. The most important functional distinction is not simply full-length versus truncated, but the presence or absence of the ODD, LZIP, N-TAD, and C-terminal sequence. Long ODD-containing isoforms can be subjected to VHL-dependent turnover and can retain transcriptional activity, whereas ODD-lacking isoforms such as HIF-3α4/IPAS are stable dominant-negative competitors for ARNT or HIF-1α. Because many diseases studies do not resolve these isoforms, the tables in this review assign an evidence level to each observation.

### 4.1 Mouse *Hif3a*

The mouse *Hif3a* gene is located on chromosome 7 and includes 15 exons encoding a canonical protein of 662 amino acids. Alternative splicing and promoter usage generate three major isoforms, namely full-length HIF-3α, neonatal/embryonic PAS protein (NEPAS), and inhibitory PAS protein (IPAS) [69], [70], (Fig. 1)

Mouse HIF-3α is considered the canonical full-length isoform, being 662 amino acids long. It resembles the full-length human HIF-3α1 isoform, containing bHLH, PAS-A, PAS-B, PAC, ODD, N-TAD and LZIP domains [71], [72]. Contrary to earlier assumptions, mHIF-3α is hypoxia-inducible at both mRNA and protein levels, with expression peaking after 2 h of hypoxia (1% O_2_) [73]. The mHIF-3α is widely expressed in adult mouse tissues, including lung, heart, kidney, cerebral cortex, and hippocampus**.** In lung development, mouse HIF-3α plays a critical role in alveolar formation and epithelial differentiation. Transgenic overexpression of mouse HIF-3α leads to aberrant alveolar structure characterized by reduced alveolar type I/II cells and increased aberrant basal cells expressing SOX2/p63. Further, mHIF-3α upregulated expression of proximal lung markers (e.g., Foxp2, Rarβ) and suppressed distal markers, indicating its role in modulating epithelial differentiation. Notably, mouse HIF-3α specifically suppresses HIF-2α (but not HIF-1α) levels in the developing lung, suggesting isoform-specific crosstalk in hypoxia signaling during organogenesis [70]. Additional mouse roles include cardiac protection, where HIF-3α knockout impairs tolerance to chronic hypoxia, leading to right ventricular hypertrophy and pulmonary hypertension [69], [74].

NEPAS is an isoform of HIF-3α generated through alternative mRNA splicing which replaces exon 1 b in mouse HIF-3α with an alternative exon 1a. The cDNA analysis of NEPAS indicates that its mRNA contains a unique open reading frame consisting of 1992 nucleotides, which translates into a polypeptide chain of 664 amino acids. NEPAS comprises bHLH, PAS-A, PAS-B, PAC, ODD, N-TAD and LZIP domains, closely resembling the human HIF-3α9 isoform but differing in eight N-terminal amino acids [69], [70].

Like other HIF-3α isoforms, NEPAS is hypoxia-inducible in mice and regulated by HIF-1α but not HIF-2α [70]. It is exclusively expressed during the late embryonic and early postnatal stages in embryonic heart, kidney, lung, brain, and liver tissues linking it to neonatal and embryonic development [14]. Functionally, NEPAS acts as a negative regulator of HIF-1α and HIF-2α by competing for the common partner ARNT (HIF-1β) blocking hypoxia-response element (HRE) binding, thereby suppressing HIF target genes.

NEPAS knockout mice are characterized by cardiac abnormalities such as significant enlargement of the right ventricle (RV) and right atrium (RA), reduced RV blood pressure, progressive cardiac remodeling and impaired lung remodeling due to excessive endothelial cell proliferation. Further, this phenotype is driven by upregulated endothelin-1 (ET-1), a key mediator of vascular development [69].

IPAS (inhibitory PAS domain protein) is a truncated splice variant of mouse HIF-3α that lacks the LZIP, ODD and TAD domains and retains only the bHLH, PAS-A, PAS-B, and PAC domains. Like NEPAS, IPAS is hypoxia-inducible and predominantly expressed in adult tissues, predominantly in corneal epithelium and to a lesser extend in the Purkinje cells of the cerebellum and cerebral neurons [74]. It is supposed to act as a negative regulator of the hypoxic response by binding directly to HIF-1α, forming a non-functional complex that prevents HIF-1α from binding to HREs on DNA. Additionally, IPAS competes with HIF-1α and HIF-2α for dimerization with HIF-1β, effectively blocking the transcriptional activation of HIF target genes like VEGF and PGK1 [7], [75], [76] (Table 1).

**Table 1:** Side-by-side comparison between mouse and human HIF-3α isoforms.

Isoform (mouse)	Length (aa)	Key domains	pVHL target	Expression pattern	Regulation	Primary function(s)	References
Canonical HIF-3α	664	bHLH, PAS-A/B, PAC, ODDD, LZIP (no C-TAD)	Yes	Adult: highly expressed in lung; also in heart, kidney, and brain regions (e.g. cortex, hippocampus)	Hypoxia-inducible (strong transcriptional upregulation; increased protein stability	Promotes alveolar/distal epithelial differentiation and protection from hypoxic injury; can antagonize HIF-2α-dependent programs; contributes to cardioprotection and, via targets such as GPx4, to ferroptosis resistancee	[15], [77], [78], [79]
NEPAS	664; Identical to full-length HIF-3α except for first 8 N-terminal aa	bHLH, PAS-A/B, PAC, ODDD, LZIP (8 aa N-term difference)	Yes	Predominantly embryonic/neonatal: heart, lung, kidney, brain, liver	Hypoxia-inducible; transcriptionally regulated by HIF-1α via HREs in the Hif-3α locus	Context-dependent regulator: can act as dominant-negative by competing with HIF-1/2α for ARNT, but also functions as an oxygen-regulated transcription factor with a distinct target set	[69,85,91]
IPAS	Truncated (∼400)	bHLH, PAS-A/B, PAC (lacks ODD, LZIP, C-TAD)	No	Adult mouse: cornea, cerebellum, select neuronal populations” to signal species and avoid implying pan-neuronal expression	Tissue-specific; hypoxia-inducible in several contexts	Dominant-negative: forms non–DNA-binding dimers with HIF-1α (and in some contexts HIF-2α), displacing ARNT and repressing HIF-dependent genes (e.g. VEGF, PGK1)	[80], [81]
HIF-3α1 (canonical)	669	bHLH, PAS-A/B, PAC, ODD, N-TAD, LZIP (no C-TAD)	Yes	Broad adult + embryonic	Hypoxia-inducible	Transcriptional activator of LC3C, REDD1, SQRDL; confers GPx4-dependent protection from ferroptosis in lung models; subject to AKT-dependent phosphorylation at Ser524	[79], [82], [83]
HIF-3α9	669	Identical to HIF-3α1 (differs by 8 N-terminal aa)	Yes	Broad adult + embryonic	Hypoxia-inducible; oleoylethanolamide (OEA) lipid sensing	Metabolic sensor via PAS-B binding of OEA. OEA-bound HIF-3α9 enhances heterodimer stability and influences proliferation and migration in a context-dependent manner.	[77,85,94]
HIF-3α2	632	bHLH, PAS-A/B, PAC, ODD, N-TAD (incomplete LZIP, no second LXXLL)	Yes	Adult + embryonic (e.g., liver, brain)	Hypoxia-inducible but oxygen-insensitive protein stability	Transcriptional activator that can induces EPO in hepatoma and neuroblastoma cells; Acts as hypoxia-inducible “timer” regulating VEGFA, GLUT1, DDIT4 and caspase-3 and influences endothelial fate and vascular remodeling. Some evidence for Wnt/β-catenin suppression in specific contexts.	[84], [85], [86]
HIF-3α4 (IPAS-like)	∼363	bHLH, PAS-A/B, PAC (lacks ODD, LZIP, TAD; retains full PAS-B)	No	Cornea, brain, vasculature	Hypoxia-inducible	Expressed in cornea, brain, vasculature. Dominant-negative: competes for ARNT or binds HIF-1α; inhibits VEGF, PGK1	[15,77,85,92]
HIF-3α5/HIF-3α6	Variable	Short C-terminally truncated variants; predicted bHLH/PAS but incomplete ODD/TAD; some only described at mRNA level	Unknown	Limited data	—	Not experimentally defined; potential inhibitory roles are hypothetical	[15]
HIF-3α7/HIF-3α8	Variable	PAS-A/B, PAC, C-terminally divergent; ODD presence isoform-dependent	Unknown, if yes, only in ODD-containing variant	Pancreas-specific (HIF-3α7); HIF-3α8: limited data	Hypoxia-inducible	Poorly characterized; likely non-canonical or tissue-specific regulatory roles	[71,85]
HIF-3α10	7	Truncated peptide (retained intron → premature stop)	No	—	—	Likely non-coding transcript; no protein function demonstrated	[[15], [71], [87]]

A side-by-side comparison of mouse and human HIF-3α isoforms, including domain composition, VHL sensitivity, and dominant -negative properties, is provided in Table 1.

### 4.2 Human *HIF3A*

The human *HIF3A* gene spans approximately 43 kilobases on chromosome 19q13.2 and contains 19 exons that generate 19 computationally predicted splice variants through three alternative transcription start sites (exons 1a, 1 b, 1c) and alternative exons 13/14. From the predicted protein-coding isoforms (HIF3a1–10), full-length cDNA could be obtained for the majority [14], [88]. This remarkable isoform diversity-contrasting with the three major isoforms in mice-underlies HIF-3α′s context-specific functions across development, metabolism, and disease [89] (Table 1).

#### 4.2.1 Full-length *isoforms* (transcriptionally active)

Critically, HIF-3α1/HIF-3α9 lack a classical C-terminal transactivation domain (C-TAD), rendering them relatively resistant to factor-inhibiting HIF (FIH)-mediated asparaginyl hydroxylation and enabling sustained transcriptional activity under hypoxia [88].

The LZIP domain, unique to HIF-3α1/HIF-3α9, mediates crucial protein-protein interactions and transcriptional regulation enabling non-canonical target gene regulation. Contrary to early views positioning HIF-3α solely as a hypoxia response inhibitor, full-length isoforms actively regulate target genes. Specifically, HIF-3α1/HIF-3α9 upregulate SQRDL (sulfide:quinone oxidoreductase) and REDD1 (regulated in development and DNA damage response 1) through non-canonical hypoxia-response elements (HREs). This expands the transcriptional repertoire of HIF-3α beyond the classical HIF pathways. Additionally, insulin signaling via PKB/AKT phosphorylates HIF-3α1 at serine 524 under normoxic conditions, promoting cell proliferation and migration [82].

Studies from primary hepatocytes and rat livers support this view as rat HIF 3α, like HIF 1α and HIF 2α, can directly stimulate HRE-dependent transcription [90]. In perivenous zones of rat liver lobules [82], HIF-3α mRNA is enriched alongside HIF-1α and HIF-2α mRNA, suggesting that all three α-subunits contribute to zonated hypoxic responses in hepatocytes [90]. Furthermore, rat HIF-3α variants have been shown to positively regulate the promoter activity of insulin-like growth factor-binding protein 1 (IGFBP-1b), supporting a broader transcriptional activator role for HIF-3α in regulating metabolic targets in liver [91]. Similarly, studies in zebrafish have shown that Hif-3α functions as an oxygen-dependent transcriptional activator by binding directly to the promoters of a unique set of target genes, including igfbp-1b, redd1, sqrdl, and mclb [83].

#### 4.2.2 Truncated isoforms

HIF-3α2 (632 aa) structurally resembles full-length isoforms but lacks the second LXXLL motif and complete LZIP domain [88]. Expressed across adult and embryonic tissues regardless of oxygen tension, HIF-3α2 operates via oxygen-insensitive mechanisms yet retains robust HRE-dependent transcriptional activity [89]. HIF-3α2 directly suppresses Wnt/β-catenin signaling by destabilizing nuclear β-catenin complexes. In human umbilical vein endothelial cells (HUVECs), HIF-3α2 accumulates during prolonged hypoxia through transcriptional upregulation and protein stabilization, regulating endothelial fate and vascular remodeling [84].

Apart from the dominant negative action of HIF-3α2 on Wnt/β-catenin signaling, HIF-3α2 was shown to upregulate erythropoietin (EPO) expression by binding together with ARNT canonical HREs in the EPO promoter and 5′ flanking regions in Hep3B and SK-N-AS cells [85]. Overexpression of HIF-3α2 increased EPO mRNA by approximately 5- to 7-fold and EPO protein by 6- to 7-fold, whereas siRNA targeting the shared HIF3A mRNA region reduced EPO production without altering *HIF1A* or *EPAS*1 gene expression [85].

HIF-3α4 (363 aa), a key truncated isoform, retains bHLH, PAS-A, and PAS-B domains but lacks both ODD and LZIP domains, functioning as a dominant-negative inhibitor structurally analogous to mouse IPAS [88]. HIF-3α4 competes with HIF-1α/HIF-2α for HIF-1β (ARNT) binding, suppressing transcriptional activation of metabolism and angiogenesis genes such as VEGF [85,86]. Paradoxically, HIF-3α4 stabilizes HIF-1α in renal carcinoma cells, promoting tumor growth and demonstrating context-dependent enhancement of hypoxia signaling [92].

#### 4.2.3 Specialized and *tissue*-specific isoforms

HIF-3α9, beyond its structural similarity to HIF-3α1, serves as a metabolic sensor: oleoylethanolamide (OEA)-a lipid-derived regulator of food intake-selectively binds HIF-3α9, enhancing stability and modulation of the HIF-3α/ARNT heterodimer [93]. Unsaturated fatty acids also act as high-affinity PAS-domain ligands, enabling direct sensing of cellular lipid metabolism status [93]. HIF-3α7 exhibits pancreas-specific expression, while HIF-3α10 contains a retained intron in exon 1, producing a premature stop codon that renders it non-functional [88]. Additional short variants (HIF-3α5/6) inhibit HIF-1α/HIF-2α via ARNT sequestration and HRE competition [15].

## 5 Regulation of HIF3A: transcriptional, epigenetic, post-transcriptional, post-translational, and metabolic control

HIF3A is regulated at multiple levels. At the transcriptional level, HIF3A is induced by hypoxia and is a target of HIF-1α in several systems. At epigenetic level, promoter methylation of HIF3A is altered in prostate cancer, breast cancer, obesity-related liver dysfunction, and other settings. At the post-transcriptional level, miRNAs and lncRNAs can control total HIF3A abundance or shift the balance between isoforms. It was shown that hypoxia-induced miR-429 directly regulates HIF3A2 and HIF3A3, and miRNA-mediated regulation has been proposed to facilitate the transition from early HIF-1α-dependent responses to later HIF-3α-dependent adaptation [94]**.** Further, HIF-3α isoforms are regulated in a cell type-specific and hypoxia-dependent manner in vascular cells, supporting the idea that the local isoform composition, rather than a single HIF-3α protein, determines the vascular response [92]. HIF-3α2 also accumulates during prolonged hypoxia in endothelial cells and regulates endothelial fate, which supports a temporal role for long HIF-3α isoforms in chronic hypoxia. Additional miRNAs, including miR-184, miR-300, miR-485-5p, and miR-210-3p, have been linked to HIF3A control in cancer or splicing contexts [95], [96], [97], although the isoform specificity of several of these interactions remains to be defined. At post-translational, AKT/PKB phosphorylates HIF-3α1 at Ser524 in response to insulin [82], and OEA binding to HIF-3α9 couples the protein to lipid metabolism [98]. These regulatory layers explain why total HIF3A expression can be misleading and why isoform-resolved assays are required.

## 6 Redox regulation of HIF-3α: established, indirect, and proposed mechanisms

The redox dimension of HIF-3α biology should be divided into three categories. The first category contains experimentally supported HIF-3α functions that alter redox outcomes. The second category contains indirect associations in which HIF-3α expression or activity changes in redox-stressed tissues, but the causal direction is not fully established. The third category contains plausible mechanisms that have not yet been demonstrated for HIF-3α (Table 2).

**Table 2:** Current evidence, mechanistic gaps, and experimental priorities in HIF-3α redox biology.

Category	Mechanism	Current status	What would be needed
Established redox-related output	HIF-3α increases GPx4 and limits ferroptosis in lung epithelial cells	Supported by mouse genetic and pharmacologic data and human COPD expression correlation	Human isoform specific perturbation
Established non-redox regulatory input	OEA binds HIF-3α9 and AKT/PKB phosphorylates HIF-3α1	Direct biochemical or functional evidence	Link to redox state in primary human cells
Indirect association	HIF-3α expression changes in oxidative, hypoxic, metabolic, or tumor tissue	Correlative	Causal isoform-specific experiments
Proposed direct redox modification	ROS, NO, or lipid peroxidation products modify HIF-3α cysteines or LZIP	Not demonstrated	Redox proteomics, cysteine mutagenesis, dimerization and DNA binding assays
Proposed PHD or FIH control	PHD or FIH hydroxylation regulates HIF-3α stability or transactivation	Not established with HIF-3α-specific rigor	Hydroxylation site mapping, PHD or FIH inhibition, isoform-specific stability assays

**Established redox-related functions.** The strongest current evidence is the HIF-3α-GPx4 axis in lung epithelial cells. In mouse models of cigarette smoke exposure, canonical HIF-3α increases GPx4, reduces lipid peroxidation, and protects against ferroptosis [79]. Human COPD data show a concordant HIF-3α-GPx4 expression signature and association with lung function [79]. These studies establish that HIF-3α can regulate a redox-protective transcriptional program, although they do not yet prove that ROS directly modify HIF-3α.

**Indirect redox associations.** HIF-3α expression is altered in oxidative or hypoxic disease settings, including COPD, obesity-related liver dysfunction, neurodegeneration, and cancer. OEA binding to HIF-3α9 links HIF-3α activity to metabolism, and lipid peroxidation products can influence cellular redox state. AKT-dependent phosphorylation of HIF-3α1 connects growth-factor signaling to HIF-3α function. These observations are compatible with redox regulation, but they do not by themselves demonstrate direct redox control of HIF-3α.

**Proposed but unproven mechanisms.** By analogy with HIF-1α and HIF-2α, ROS could stabilize HIF-3α by inhibiting PHDs, and cysteine oxidation, S-nitrosylation, or S-glutathionylation could alter HIF-3α dimerization, nuclear retention, or co-regulator recruitment. The LZIP domain is a plausible site for redox-sensitive dimerization, but no HIF-3α-specific cysteine redox switch has been demonstrated. Likewise, direct PHD or FIH hydroxylation of HIF-3α has not been established with the same rigor as for HIF-1α and HIF-2α. Convergence with NRF2 through GPx4 is supported by the lung data but remains a pathway-level association rather than proof of direct HIF-3α-NRF2 interaction. Therefore, statements that HIF-3α is a redox sensor should be qualified as model-based until proteomic, mutagenesis, and isoform-specific functional data are available.

## 7 An integrative model section

The available data support a model in which HIF-3α isoforms function as context-dependent rheostat rather than as a single dominant-negative protein. During acute hypoxia, HIF-1α dominates glycolytic and survival responses. During prolonged hypoxia, HIF-3α isoforms can accumulate and reshape the response. Long isoforms such as HIF-3α1, HIF-3α2, and HIF-3α9 can activate selected targets, including EPO, LC3C, REDD1, SQRLD, IGFBP-1, and GPx4 in specific tissues. Short isoforms such as HIF-3α4/IPAS can limit HIF1/2-dependent angiogenic and metabolic programs. The net outcome depends on four variables: the dominant isoform, the cell type, the oxygen and redox history, and the epigenetic or miRNA state of the *HIF3A* locus. This model explains several apparent contradictions. A tumor with high HIF-3α1 may be less angiogenic because HIF-3α4 sequesters ARNT or HIF-1α. A tumor with HIF-3α4 in a HIF-2α-driven context may behave differently if the isoform stabilizes or redirects HIF-2α. The same logic applies to lung, heart, liver, and brain, where HIF-3α can be protective when it supports GPx4-dependent redox defense and pathogenic when it sustains proliferative or inflammatory programs. This also supports the hypothesis that HIF-3α is a temporal regulator of chronic hypoxia, but this hypothesis requires time-resolved isoform profiling.

## 8 HIF-3α in disease contexts

### 8.1 Mouse HIF-3α pathological roles

Mouse *Hif3a* produces three major isoforms: canonical HIF-3α, NEPAS, and IPAS [15], [69], [88]. HIF-3α is hypoxia-inducible in mice, with expression peaking within a few hours at low oxygen tensions (around 1% O_2_) through mRNA upregulation and protein stabilization, and is found in lung, heart, kidney, cortex, and hippocampus [99], [100]. While mouse isoforms are far less diverse than the 8–10 functional human variants, their core structural domains parallel human HIF-3α1, enabling conserved hypoxia-responsive functions [88]. In lung development, HIF-3α overexpression causes distal alveolar defects, including reduced type I/II pneumocytes, expansion of SOX2^+^/p63^+^ basal-like cells, proximalization of gene expression (Foxp2, Rarβ), and selective downregulation of HIF-2α but not HIF-1α [70]. NEPAS, expressed predominantly in late embryonic and early postnatal stages, supports developmental hypoxia responses [15], [69]. IPAS inhibits HIF-1-mediated apoptosis in brain during severe hypoxia, acting as a protective dominant-negative isoform.

In cardiac physiology, *Hif3a* knockout mice show impaired tolerance to chronic hypoxia, including right ventricular hypertrophy and pulmonary hypertension, implicating canonical HIF-3α isoforms in cardiopulmonary adaptation to low oxygen [69], [74]. In metabolic settings, mouse models with *Hif3a* deficiency exhibit impaired glucose tolerance and insulin sensitivity, likely mediated by canonical isoforms analogous to human HIF-3α1/HIF-3α9, linking HIF-3α to systemic metabolic homeostasis [74].

In ferroptosis regulation during cigarette smoke-induced lung injury, mouse canonical HIF-3α (structurally closest to human HIF-3α1) suppresses cell death via GPx4 activation. In MLE-12 cells exposed to 5% cigarette smoke extract (CSE) for 24 h, HIF-3α knockdown elevates ROS (DCFH-DA), increases lipid peroxidation (C11-BODIPY), induces mitochondrial abnormalities (smaller mitochondria, dense membranes, cristae loss), and reduces SOD activity and cell viability (CCK-8), whereas HIF-3α overexpression reverses these changes through upregulation of GPx4 mRNA and protein [79]. These protective effects are abrogated by RSL3 (0.1 μM), a GPx4 inhibitor, confirming GPx4 dependence [79]. RNA-seq/GSEA reveals ferroptosis pathway enrichment (NES > 1, p < 0.05, FDR < 0.25) in HIF-3α-modulated cells, indicating a consistent transcriptomic ferroptosis signature [79].

In vivo, Sftpc^CreERT2/+ R26^LSL-Hif3a mice with tamoxifen-induced HIF-3α overexpression in alveolar type II cells exposed to chronic cigarette smoke (≈160–180 mg/m^3^ TPM, 4 h/day, 5 days/week, 6 months) maintain lung function (higher FEV0.05/FVC by FlexiVent), show preserved alveolar structure (lower MLI, higher MAN), increased GPx4 expression (immunofluorescence and Western blot), and reduced oxidative and ferroptotic markers (DHE, 4-HNE, TUNEL) relative to control genotypes [79]. These findings position mouse canonical HIF-3α-most similar to human full-length HIF-3α1/HIF-3α9-as an *anti*-ferroptotic, tissue-protective factor in oxidative lung injury, functionally distinct from HIF-1α and HIF-2α in this setting [79].

## 9 Human HIF-3α: pathological roles

### 9.1 Human HIF-3α in metabolic disease and redox regulation

Full-length isoforms HIF-3α1 and HIF-3α9 rs3826795 methylation correlates with elevated alanine aminotransferase (ALT) levels in obese children, linking HIF-3α epigenetic regulation to liver dysfunction in obesity [101]. In human and mouse adipose-related contexts, knockdown of full-length HIF-3α isoforms has been associated with reduced expression of target genes such as ANGPTL4 and circulating ANGPTL4 levels inversely correlate with obesity in monozygotic twins, suggesting that these isoforms influence lipid metabolism and hepatic/adipose cross-talk—potentially via HIF-3α9's OEA/lipid sensing capacity [74], [93], [101]. In diabetes, mouse data show that reduced HIF-3α impairs glucose tolerance and insulin sensitivity, implying a conserved role for HIF-3α1/HIF-3α9-like full-length isoforms in glucose homeostasis; however, direct human functional validation of this axis remains limited [74].

At the redox level, full-length isoforms regulate ferroptosis-an iron-dependent cell death pathway involving lipid peroxidation, glutathione (GSH) depletion, and mitochondrial damage—primarily through transcriptional activation of glutathione peroxidase 4 (GPx4), which reduces lipid hydroperoxides to alcohols using GSH, thereby mitigating ROS accumulation and membrane rupture. This HIF-3α1/HIF-3α9–GPx4 axis is hypoxia-inducible and protective against oxidative stress, as evidenced in COPD models where cigarette smoke induces early ferroptosis in the alveolar epithelium [79]. Mouse studies provide detailed mechanistic insights via genetic and pharmacologic manipulation of canonical isoforms (analogous to human HIF-3α1), whereas human data are currently based on expression correlations and pathway analysis, reflecting the practical difficulty of directly manipulating specific HIF-3α isoforms in human tissues.

The metabolic data should be interpreted as evidence for a full-length HIF-3α lipid and glucose sensor, but the human functional evidence remains correlative. The OEA-HIF-3α9 interaction provides the most direct molecular link to lipid sensing, whereas the GPx4 axis provides the most direct redox link.

### 9.2 Human HIF-3α in lung disease

In lung disease, full-length HIF-3α isoforms (HIF-3α1/HIF-3α9) emerge as key regulators of redox balance and cell death via the GPx4 axis. In COPD, bulk RNA-seq of 36 lungs (non-smokers, smokers, pre-COPD, COPD) and Mfuzz clustering place HIF-3α in a redox-enriched gene cluster, with expression positively correlating with FEV1/FVC [79]. Downregulation of HIF-3α1/HIF-3α9 in pre-COPD and COPD coincides with upregulation of ferroptosis-related signatures, suggesting early vulnerability to ferroptotic damage in the alveolar compartment. Single-cell RNA-seq (GSE171541) localizes HIF-3α transcripts predominantly to alveolar epithelial (EpCAM^+^) cells rather than airway epithelium, consistent with the alveolar-focused protection observed in mouse canonical HIF-3α models [79]. Although no direct human mechanistic perturbation of specific isoforms has yet been performed, the concordance between mouse functional data (canonical HIF-3α → GPx4) and human expression patterns strongly supports a conserved protective HIF-3α1/HIF-3α9–GPx4–ferroptosis axis in lung tissue [79] (Fig. 3).

**Fig. 3:**
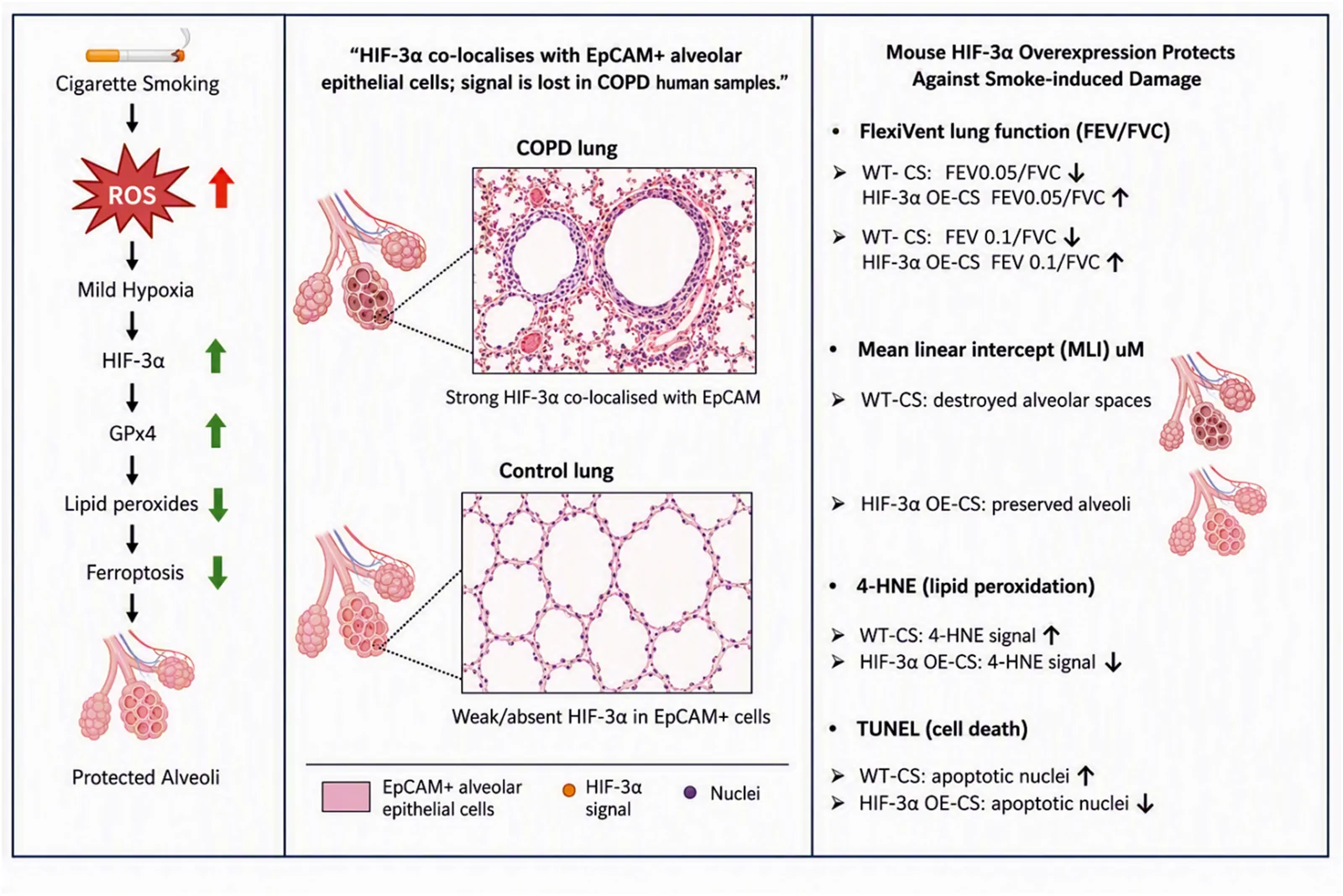
**HIF-3α protects against cigarette smoke-induced oxidative lung injury. Left:** Proposed HIF-3α-GPX4 protective axis, in which cigarette smoke-induced ROS and mild hypoxia increase HIF-3α and GPX4, limiting lipid peroxidation and ferroptosis. **Middle:** HIF-3α co-localizes with EpCAM ^+^ alveolar epithelial cells in control lung but is reduced or absent in COPD samples. **Right:** In cigarette smoke-exposed mice, HIF-3α overexpression improves lung function, preserves alveolar architecture, and reduces 4-HNE accumulation and TUNEL positivity compared with WT controls. Histological structures are schematic. **Abbreviations:** 4-HNE, 4-hydroxynonenal; COPD, chronic obstructive pulmonary disease; CS, cigarette smoke; EpCAM, epithelial cell adhesion molecule; FEV, forced expiratory volume; FVC, forced vital capacity; GPX4, glutathione peroxidase 4; HIF-3α, hypoxia-inducible factor-3α; MLI, mean linear intercept; OE, overexpression; ROS, reactive oxygen species; TUNEL, terminal deoxynucleotidyl transferase dUTP nick-end labeling; WT, wild type.

The lung section therefore supports a conserved HIF-3α-GPx4 protective axis, but it does not yet prove that HIF-3α1 or HIF-3α9 is the causative isoform. Isoform-specific perturbation in human alveolar epithelial cells is needed.

In primary spontaneous pneumothorax (PSP), elevated HIF-3α and caspase-8 expression in mesothelial cells, alveolar type II cells, and bronchoalveolar epithelial cells indicate contributions of hypoxia, inflammation, and cell death pathways to PSP pathogenesis—potentially involving dominant-negative isoforms like HIF-3α4 that modulate classical HIF responses [79]. As noted, HIF-3α1/HIF-3α9-mediated ferroptosis inhibition via GPx4 provides a mechanistic link between hypoxia-regulated transcriptional responses and structural integrity of the alveolar region in chronic smoke exposure [79].

### 9.3 Human HIF-3α in neurodegeneration

In neurodegeneration, both full-length (HIF-3α1/HIF-3α9) and inhibitory (HIF-3α4) isoforms may contribute to pathological dysregulation. In Alzheimer's disease (AD), specific m6A methylation sites within the *HIF3A* gene, particularly at position 3632, increase HIF-3α expression in brain tissue. This reduction in KDM3A and TGF-β1 levels occurs under circadian rhythm disruption and forms a regulatory axis that may contribute to AD progression. This process likely involves full-length isoforms with intact transactivation capacity [102]. In amyotrophic lateral sclerosis (ALS), differential HIF-3α expression in induced pluripotent stem cell (iPSC)-derived motor neurons and fibroblasts from patients compared to healthy controls is associated with mitochondrial dysfunction, tissue hypoxia, and oxidative stress. This can potentially impair neuroprotective responses, and the balance of activating and inhibitory forms of the isoform could determine the overall redox protection [102]. While ferroptosis-specific readouts (e.g., GPx4-dependent lipid peroxidation via HIF-3α1/HIF-3α9) have not been systematically analyzed in these contexts, the intersection of hypoxia, reactive oxygen species (ROS), and HIF-3α dysregulation suggests that ferroptotic stress may converge with isoform-specific HIF-3α signaling in the nervous system [102].

The neurodegeneration data are currently correlative and isoform-unresolved. They support a model in which HIF-3α dysregulation intersects with m6A modification, circadian control, mitochondrial dysfunction, and oxidative stress, but they do not yet establish a specific HIF-3α isoform as causal.

## 10 Human HIF-3α in cancer

Cancer studies on HIF-3α must be interpreted with caution because many reports measure total HIF3A mRNA, total HIF-3α protein, or immunohistochemical staining without defining the responsible isoform. The most reliable conclusions come from studies that manipulate a defined isoform or show isoform-specific expression. HIF-3α1 has the strongest isoform-resolved tumor-promoting evidence in colorectal cancer, where it activates JAK-STAT3, promotes EMT, and increases iron uptake. HIF-3α4/IPAS has the strongest isoform-resolved tumor-suppressive evidence in meningioma and in some renal cell carcinoma settings, where it limits HIF-1-dependent angiogenesis and metabolism. However, HIF-3α4 can also interact with HIF-2α in a way that may support HIF-2-driven growth, so even the truncated isoform is context-dependent. Studies based on total HIF-3α expression, promoter methylation, or miRNA targeting are valuable but should be classified as correlative or functional only when the isoform is defined (Table 3).

**Table 3:** HIF-3α in selected human cancers.

Cancer type	Dominant signal and isoform resolution	Evidence level	Key mechanisms and regulators	Prognostic association	References
Colorectal carcinoma (CRC)	HIF-3α1 ↑ (full-length, LZIP-containing); full-length HIF-3α1 specifically identified	Direct mechanistic and functional validation, with correlative survival data	Non-canonical JAK–STAT3 activation via cytosolic scaffolding; EMT via ZEB2; iron loading via TFRC; increased HRE reporter activity and survival under oxidative stress	High HIF-3α1 associated with poor survival in multiple GEO datasets; knockdown reduces growth and stress resistance	[103], [104]
Pancreatic ductal adenocarcinoma (PDAC)	Total HIF-3α ↑ under hypoxia (small and full-length variants); functionally relevant isoform not fully resolved	Functional validation in cell lines, correlative clinical association	Preferential hypoxic induction vs HIF-1α/2α; direct transcriptional activation of RhoC–ROCK1; promotion of proliferation, migration, invasion	High HIF-3α correlates with increased distant metastasis and reduced overall survival (∼2.8-fold higher metastatic risk)	[105]
Prostate cancer	*HIF3A* hypermethylation (↓ full-length isoforms); individual isoform not resolved	Correlative clinical association	Promoter CpG hypermethylation (Marmal-Aid screen); inverse correlation between methylation and expression	Hypermethylation strongly associated with biochemical recurrence after radical prostatectomy (AUC ∼0.8)	[106]
Breast cancer	*HIF3A* hypermethylation, HIF-3α ↓; individual isoform not resolved	Correlative clinical association	Elevated CpG methylation in tumors and blood; links to obesity and weight gain	Higher *HIF3A* methylation associates with increased breast cancer risk in Mexican American women	[107]
NSCLC	Truncated HIF-3α (HIF-3α4/IPAS-like) and miR-300–HIF-3α axis; relevant isoform not fully resolved	Functional validation and correlative association	miR-300 directly targets *HIF3A*; HIF-3α knockdown and miR-300 overexpression suppress proliferation and metastasis	Stage-dependent: early HIF-3α elevation predicts progression, while miR-300–mediated suppression reduces metastasis	[97]
Meningioma	HIF-3α4/IPAS ↑; isoform specifically identified	Isoform-resolved correlative functional evidence	Dominant-negative inhibition of HIF-1/2 targets; reduced angiogenesis and glycolysis	Higher IPAS/HIF-3α4 expression correlates with lower proliferation and microvessel density	[108], [109]
Osteosarcoma	Total HIF-3α ↑; individual isoform not resolved	Functional validation and correlative association	SOX9 promoter demethylation via KDM3A; activation of JAK2–STAT3 pathway	Associated with tumor progression and poor prognosis	[108], [110]
Gastric adenocarcinoma	miR-184–mediated HIF-3α suppression; individual isoform not resolved	Functional validation	miR-184 targets *HIF3A*, reducing HIF-3α and enhancing glycolysis and invasion; restoring HIF-3α dampens these effects	High miR-184/low HIF-3α associated with aggressive disease	[95], [108]
Ovarian cancer	HIF-3α ↑ via lncRNA LINC01342; individual isoform not resolved	Functional validation, correlative association	LINC01342 upregulates HIF-3α, promoting migration and clonogenicity	Elevated HIF-3α associated with higher disease progression	[111]
Cervical cancer	Total HIF-3α ↑; individual isoform not resolved	Correlative association	Promotion of EMT, radioresistance, and resistance to apoptosis (isoforms not resolved)	Elevated HIF-3α associated with poor treatment response and outcome	[108]
Renal cell carcinoma (RCC)	HIF-3α4/IPAS variants (context-dependent); isoform specifically identified	Isoform-resolved mechanistic and functional evidence, context-dependent	Formation of abortive complexes with HIF-2α; can stabilize and enhance HIF-2 activity	May contribute to growth in HIF-2-driven RCC despite generally dominant-negative properties	[92], [112]

In colorectal cancer (CRC), full-length HIF-3α1 promotes tumor growth. Increased HIF-3α expression is associated with poor survival. Experimental overexpression or knockdown modulates growth, epithelial-mesenchymal transition (EMT), iron metabolism, and oxidative stress tolerance, as discussed above [103], [104]. Apparently, subcellular localization adds complexity. In CRC, for example, cytosolic HIF-3α1 acts as a scaffold for JAK-STAT3 signaling independently of nuclear HRE binding [104]. In contrast, in hepatocellular carcinoma, increased mitochondrial HIF-3α expression, compared with adjacent non-tumor liver tissue, suggests non-canonical mitochondrial roles in metabolism or ROS handling [113].

In pancreatic ductal adenocarcinoma (PDAC), hypoxia preferentially induces HIF-3α more strongly than HIF-1α or HIF-2α, and PDAC specimens show elevated HIF-3α expression, which correlates with distant metastasis and reduced overall survival [105]. HIF-3α overexpression in PANC-1 cells accelerates proliferation, migration, and invasion, whereas knockdown in MiaPaCa-2 cells reduces these phenotypes apparently by directly transactivating RhoC and ROCK1, driving actin cytoskeleton remodeling and metastatic behavior [105].

In prostate cancer, *HIF3A* promoter hypermethylation is tumor-specific and rarely observed in benign prostate or other malignancies [106]. *HIF3A* methylation strongly predicts biochemical recurrence after radical prostatectomy, with AUC values approaching ∼0.82, supporting an important tumor-suppressive role for HIF-3α in prostate epithelium [106].

In breast tissue, *HIF3A* is significantly hypermethylated and down-regulated in cancers, and *HIF3A* methylation in blood associates with obesity, weight gain, and a 2.7-fold increased breast cancer risk in Mexican American women [107]. Together with the prostate data, this places hormone-responsive cancers toward the “HIF-3α-deficient, poor-prognosis” corner.

In osteosarcoma, total HIF-3α upregulation promotes tumor progression by inducing SOX9 promoter demethylation in a KDM3A-dependent manner and activating JAK2–STAT3 signaling, thereby supporting proliferation and invasion [114]. In gastric adenocarcinoma, hypoxia-induced miR-184 directly targets *HIF3A*, lowering HIF-3α and thereby enhancing glycolysis and invasion; restoration of HIF-3α expression or miR-184 inhibition reduces these aggressive phenotypes, suggesting a partially tumor-suppressive role for HIF-3α in this context [95].

In cervical cancer, total HIF-3α expression is increased and has been linked to radioresistance, EMT, increased migration and invasion, and reduced apoptosis, nominating HIF-3α as a potential radiosensitization target, although isoform-level resolution is lacking [108]. In ovarian cancer, the lncRNA LINC01346 upregulates HIF-3α, promoting migration, clonogenicity, and cisplatin resistance; HIF-3α knockdown in patient-derived xenografts increases cisplatin sensitivity by approximately three-fold and reduces metastatic burden, identifying a lncRNA–HIF-3α chemoresistance axis [115].

In NSCLC, HIF-3α (largely shorter isoforms) interacts with miR-300; miR-300 overexpression reduces proliferation and xenograft growth while down-regulating HIF3A, and HIF-3α knockdown partially phenocopies these effects, defining a miR-300–HIF-3α axis that constrain malignant progression in at least some stages [97]. In meningioma, IPAS/HIF-3α4 expression correlates with lower proliferation and angiogenesis, suggesting a favorable prognostic role [109] In RCC, down-regulation of HIF-3α4/IPAS removes dominant -negative pressure on HIF-1, but IPAS-like variants can also stabilize HIF-2α and enhance growth of HIF-2-driven tumors, highlighting a context-dependent inversion [116], [117], [118] (Fig. 4).

**Fig. 4:**
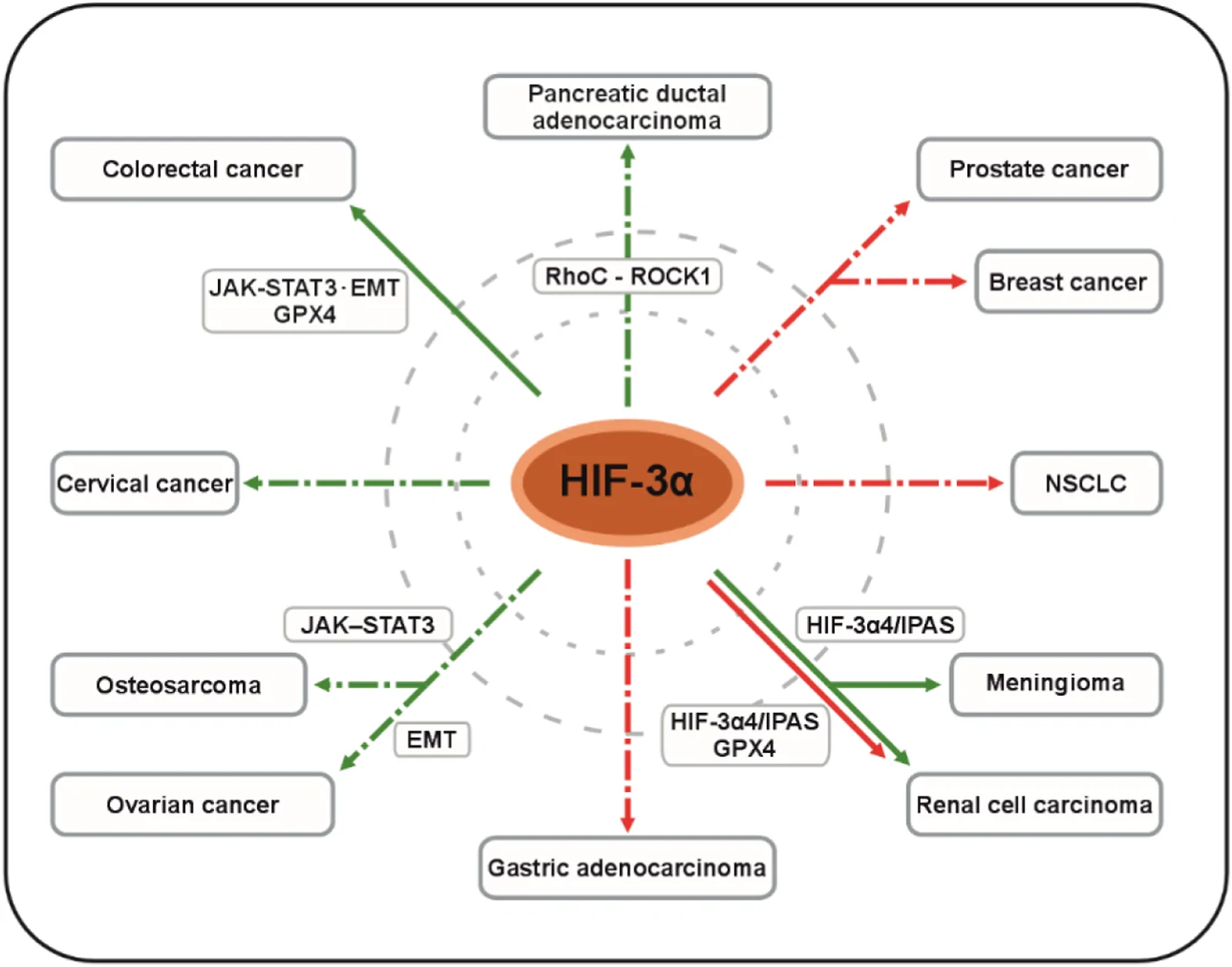
**Cancer-type-specific effects of HIF-3α isoforms and associated signaling mechanisms.** Reported effects of HIF-3α isoforms on cancer-associated signaling and phenotypes are summarized across different tumor types. Arrows point from HIF-3α toward the implicated cancers and pathways. Green arrows indicate tumor-promoting associations, whereas red arrows indicate tumor-suppressive associations. Selected mechanisms include JAK-STAT3 and RhoC-ROCK1 signaling, EMT, and GPX4-mediated redox regulation. The opposing effects reported in RCC illustrate the context- and isoform-dependent functional duality of HIF-3α4/IPAS. See text for study-specific evidence and mechanistic details. (Solid arrows representing isoform-resolved functional or mechanistic findings; dashed arrows representing total HIF3A or correlative clinical findings). **Abbreviations:** EMT, epithelial–mesenchymal transition; GPX4, glutathione peroxidase 4; IPAS, inhibitory PAS-domain protein; NSCLC, non-small-cell lung cancer.

## 11 Epigenetic and microRNA control of HIF-3α

*HIF3A* is subject to multiple layers of epigenetic regulation. In prostate cancer, aberrant promoter hypermethylation of *HIF3A* has been identified as a valuable urine biomarker, as well as for cancer recurrence. This finding has been confirmed through Marmal Aid screening and quantitative methylation-specific PCR validation [106]. In breast cancer, *HIF3A* methylation in tumors and peripheral blood associates with obesity-related weight gain and increased disease risk, suggesting systemic, metabolism-linked regulation of HIF-3α [107].

At the post-transcriptional level, multiple microRNAs and lncRNAs modulate HIF-3α. For example, miR-184 targets *HIF3A* in gastric cancer, and hypoxia-induced miR-184 represses HIF-3α, thereby increasing glycolysis and invasion [95], [119]. In the context of NSCLC, miR-300 has been shown to directly target *HIF3A*, with its overexpression resulting in the suppression of tumor growth. Conversely, the knockdown of HIF-3α has been observed to mimic the effects of miR-300, suggesting the presence of a regulatory axis between miR-300 and HIF-3α that plays a crucial role in the inhibition of malignant [95], [119]. In ovarian cancer, elevated LINC01342 expression correlates positively with HIF-3α levels, and activation of this axis enhanced the metastatic potential of cancer cells [111]. In sarcomas, hypoxia-induced miR-485-5p and miR-210-3p influence HIF-3α splicing and expression, potentially shifting the balance between full-length and truncated isoforms, although these events remain poorly characterized in primary patient samples [96], [108].

## 12 Conclusions and future directions

HIF-3α has long been considered a minor and predominantly inhibitory branch of the HIF pathway. However, accumulating evidence from various species now suggests that it is a context-dependent hypoxia sensor with transcriptional activator and dominant-negative functions. Full-length human isoforms, such as HIF-3α1, HIF-3α2, and HIF-3α9, are bona fide transcription factors that directly regulate classical HIF targets, including IGFBP-1, EPO, LC3C, REDD1, and SQRDL, in hepatocytes, endothelial cells, and other cell types [77,80,81,84,85]. In parallel, truncated IPAS-like variants, such as HIF-3α4, that retain the bHLH-PAS domains but lack the ODD domain and transactivation capacity appear to antagonize HIF-1/2-dependent programs with strong tissue specificity [75], [120].

Comparing mouse and human HIF-3α reveals significant species differences in isoform complexity. While the mouse *Hif3a* locus produces three main isoforms, the human *HIF3A* locus encodes eight to ten distinct proteins. This divergence has tangible consequences. Canonical mouse HIF-3α is a GPx4-dependent *anti*-ferroptotic factor in cigarette smoke-induced lung injury. In contrast, human cohorts reveal a protective HIF-3α-GPx4 signature in COPD and link *HIF3A* methylation to obesity, liver dysfunction, and cancer risk [74], [79], [101]. These findings strongly suggest that murine data cannot be extrapolated to human disease without isoform- and cell-type-specific analysis.

In cancer and chronic diseases, HIF-3α emerges as a double-edged regulator. Full-length isoforms promote tumor growth, EMT, and metastasis in CRC, PDAC, osteosarcoma, cervical cancer, and ovarian cancer. They also support JAK-STAT3, RhoC-ROCK1, and iron-loading pathways. Truncated HIF-3α4/IPAS, on the other hand, often restrains angiogenesis and proliferation in NSCLC and meningioma. However, RCC provides a notable exception [104], [105], [110], [120]. Epigenetic and non-coding RNA networks—including *HIF3A* hypermethylation in prostate and breast cancer, miR-184 in gastric cancer, miR-300 in NSCLC, and LINC01346 in ovarian cancer—further tune the balance between protective and pathogenic isoforms. Thus, rational therapeutic strategies for e.g.*,* CRC and PDAC include inhibiting HIF-3α1-dependent JAK-STAT3 and RhoC-ROCK1 signaling, as well as targeting HIF-3α1-driven iron metabolism with chelators such as Dp44mT [104], [105]. In osteosarcoma, cervical cancer, and ovarian cancer, interference with KDM3A/JAK2–STAT3 signaling or lncRNA LINC01342 may attenuate HIF-3α-mediated progression and chemoresistance [110], [111]. Conversely, in prostate and breast cancer, where *HIF3A* hypermethylation and downregulation correlate with recurrence and an increased risk, restoring HIF-3α expression - ideally biased toward protective isoforms-via demethylating agents or locus-specific epigenome editing might be beneficial, though this remains to be experimentally validated [106], [107]. In NSCLC and meningioma, enhancing HIF-3α4/IPAS function or mimicking its dominant-negative impact on HIF-1/2 could suppress angiogenesis and metabolic reprogramming. In RCC, however, disrupting HIF-3α4-HIF-2α complexes is necessary to prevent tumor-promoting stabilization of HIF-2α [92], [112].

Future directions should be guided by five-key questions that are amenable to experimental testing. First, which HIF-3α isoform is dominant in a given human tissue or tumor, and can long-read sequencing and isoform-specific assays define this in patient cohorts? Second, is HIF-3α directly modified by ROS, NO, or lipid peroxidation, and do such modifications alter ARNT binding, DNA binding, or target gene selection? Third, do micro RNAs such as miR-429, miR-184, miR-300, and miR-210-3p shift the HIF-3α isoform balance during chronic hypoxia? Fourth, can HIF-3α be modulated isoform-selectivity, for example by inhibiting HIF-3α1 in colorectal or pancreatic cancer, enhancing HIF-3α4 in selected angiogenic tumors, or restoring HIF-3α-GPx4 signaling in lung disease? Fifth, how do species differences in HIF3A splicing limit the translation of mouse data into human therapy (Fig. 5)?

**Fig. 5:**
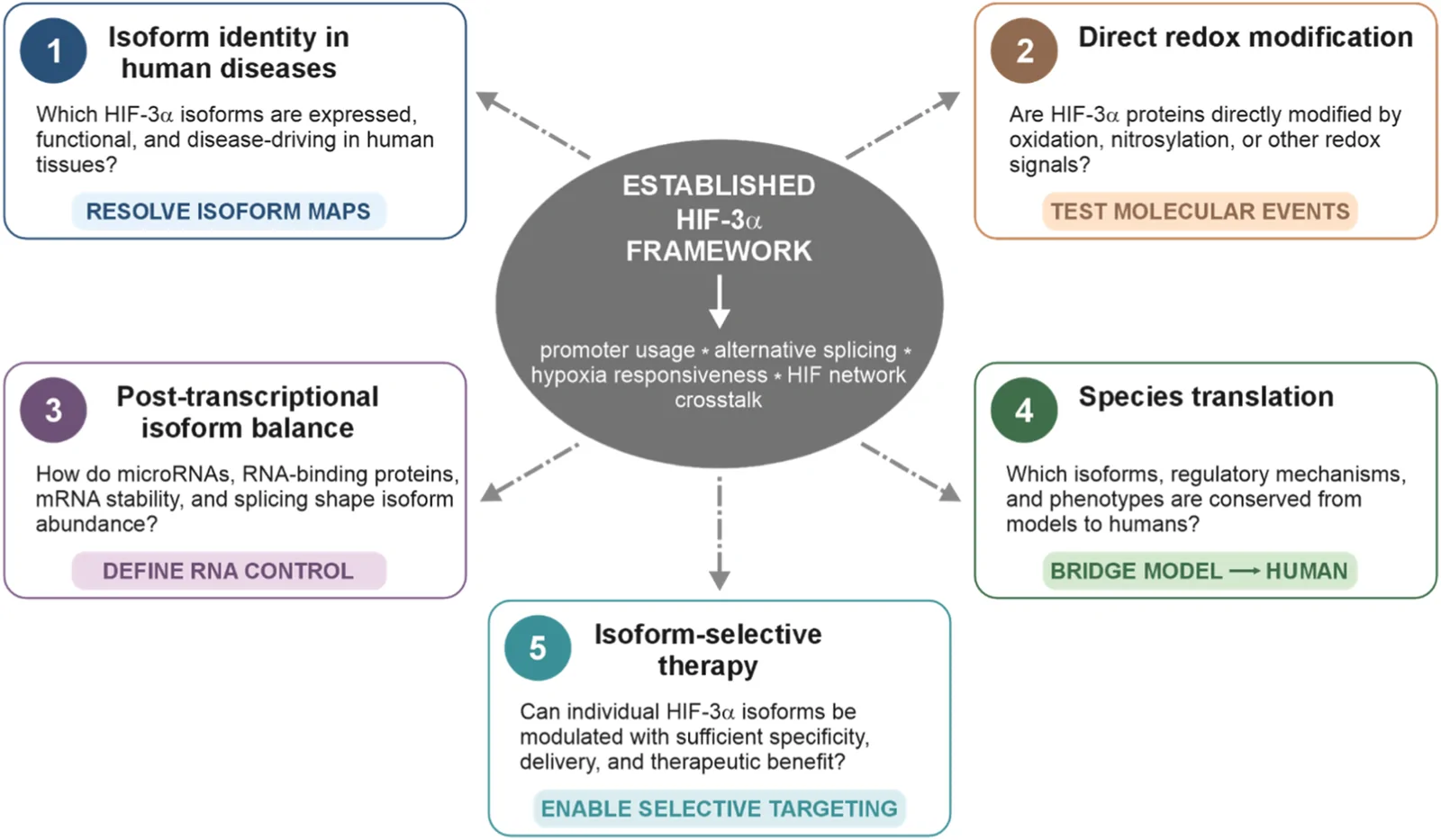
**Unanswered questions in HIF-3α biology.** Current knowledge establishes that HIF-3α is regulated through alternative promoter usage, alternative splicing and hypoxia-responsive pathways. Five major unresolved areas remain: (1) identification of disease-relevant HIF-3α isoforms in humans, (2) direct redox regulation of HIF-3α proteins, (3) post-transcriptional mechanisms controlling isoform balance, (4) translation of findings across species, and (5) development of isoform-selective therapeutic approaches.

## CRediT authorship contribution statement

**Zahra Bint e Sohail:** Writing – original draft. **Thomas Kietzmann:** Writing – review & editing, Funding acquisition. **Daniela Mennerich:** Writing – review & editing.

## Declaration of competing interest

The authors declare that they have no known competing financial interests or personal relationships that could have appeared to influence the work reported in this paper.

## Data Availability

No data was used for the research described in the article.
